# Sourdough Microbiota for Improving Bread Preservation and Safety: Main Directions and New Strategies

**DOI:** 10.3390/foods14142443

**Published:** 2025-07-11

**Authors:** Yelena Oleinikova, Alma Amangeldi, Aizada Zhaksylyk, Margarita Saubenova, Amankeldy Sadanov

**Affiliations:** 1 Research and Production Center of Microbiology and Virology, Bogenbay Batyr Str., 105, Almaty 050010, Kazakhstan; almashka91@mail.ru (A.A.); aizada_zhaksylyk@mail.ru (A.Z.); msaubenova@mail.ru (M.S.); a.sadanov1951@gmail.com (A.S.); 2 Faculty of Biology and Biotechnology, Kazakh National University Named After Al-Farabi, Al-Farabi Avenue, 71, Almaty 050040, Kazakhstan

**Keywords:** sourdough, antifungal, organic acids, antimicrobial peptides, preservation

## Abstract

Bread is consumed daily throughout the world as an important source of nutrients. However, bakery products are highly susceptible to spoilage, especially fungal, which is a source of bread losses and a threat to food security and consumer health. The use of sourdough is the best alternative to chemical preservatives, while providing a number of advantages to baked bread. This review highlights the main areas in the field of bread protection and covers the principal representatives of sourdough microbiota and their contribution to protecting bread from spoilage. The review is mainly based on publications in the field of research over the last five years, identifying new directions and strategies for bread protection related to the use of sourdoughs. A list of the main compounds produced by lactic acid bacteria of the sourdough, which contribute to the protection of bread from fungal spoilage, is presented. The contribution of other microorganisms to the antifungal effect is also considered. Finally, some prospects for the development of research in the field of sourdoughs are determined.

## 1. Introduction

Bread is an important source of nutrients and a staple food consumed daily worldwide. The average consumption of bread across European countries is 50 kg per person per year, reaching up to 80 kg per person per year in some countries [1,2]. However, the susceptibility of bread to spoilage threatens global food security. The main cause of the spoilage of bakery products is mold damage. Due to high humidity and an optimal pH range, bread and bakery products are highly susceptible to fungal spoilage, which can occur during the shelf life. Bread losses due to mold spoilage are estimated at 20% [2]. In addition to fungal growth on the surface of bread and repelling consumers with the appearance, molds cause changes in taste and produce mycotoxins, which make bread potentially unsafe. Calcium propionate has traditionally been used to inhibit mold growth in bread and bakery products. However, this additive has been shown to cause hypersensitivity, visual irritability, and attention and sleep disturbances [3].

Recently, the number of health-conscious consumers and the demand for natural and healthy products have been growing, and much attention has been paid to the safety of consumed products and the absence of chemical additives in them, which, in turn, also contributes to an increase in product spoilage. So, fungal spoilage remains a pressing problem in bread making.

The most significant agents of bread spoilage are fungi of the genera *Penicillium*, *Aspergillus*, *Paecilomyces*, and *Rhizopus* [1,4,5,6,7,8], which are characterized by a higher growth rate and competitiveness on the bread surface compared to other fungi. Well-known species of the genus *Penicillium* include *Penicillium roqueforti*, *Penicillium paneum*, *Penicillium corylophilum*, *Penicillium chrysogenum*, *Penicillium brevicompactum*, and others. The genus *Aspergillus* include *Aspergillus chevalieri*, *Aspergillus niger*, and *Aspergillus pseudoglaucus*, while *Paecilomyces variotii* is characteristic for the genus *Paecilomyces*. Bread spoilage has also been reported from the genera *Mucor*, *Endomyces*, *Cladosporium*, *Fusarium*, *Wallemia*, and *Neurospora* [5,7,9], whereas the ‘Chalk mold’ is usually caused by the yeast-like fungi *Hyphopichia burtonii* and *Saccharomycopsis fibuligera* [5].

Fungal spores present in raw materials are inactivated during baking, but can be released into the air during mixing of ingredients as aerosols and settle on finished products [5]. Fungi can also get into products during the slicing and packaging stages from the air and via equipment surfaces. Fungal spores are ubiquitous in the atmosphere at up to 1000 spores per m^3^, but in bakery air, the number of spores can be much higher.

Although the fungal spores do not survive baking, mycotoxins may remain in bread as they are relatively heat stable. Levels of mycotoxins such as aflatoxin and ochratoxin, as well as deoxynivalenol and zearalenone, are regulated by Commission Regulation (EC) No. 1881/2006. However, maximum levels for many other toxins have not yet been established, and the potential synergistic effects of different mycotoxins are also of great concern [2]. The particular problem is the intensified growth of mold in countries with higher humidity and temperatures, which requires greater attention to fungal spoilage in Asia and Africa.

Bacterial ropy spoilage of bread, characterized by sticky texture, color, and odor changes, is caused by the presence in flour of endophytic commensal wheat microbiota of the genus *Bacillus* or close genera that form heat-stable endospores [10]. The *Bacillus* species causing ropiness mainly include *B. subtilis*, *B. licheniformis*, *B. amyloliquefaciens*, and *B. pumilus*. However, spoilage of bread by other species of this and related genera of spore-forming bacteria has also been reported. It is believed that the increasing demand for preservative-free whole grain products, along with global warming, may contribute to the spread of bread ropy spoilage [10].

The high demand for natural products with a clean label is driving the desire to replace chemical preservatives with natural alternatives. Microbial fermentation is the most environmentally friendly way to preserve food and a significant substitute for chemical preservatives. Lactic acid bacteria (LAB) have long been used and studied as an alternative to chemical preservatives. LAB not only guarantee a clean label for products but also provide many benefits, such as improved sensory indicators, texture, and nutritional value. LAB have the status of GRAS (Generally Regarded as Safe) in the USA and are included in the list of Qualified Presumption of Safety (QPS) of the European Union [11].

Review papers in the field of improving the preservation and safety of bread by using sourdoughs summarize data on the dominant microorganisms of spontaneous sourdoughs, their mechanisms of spoilage control, and the effect on extending the shelf life of various types of bread. The presented review combines information on bread sourdough microorganisms, both traditional and previously unstudied in detail, but promising for use. It includes taxonomic affiliation, production of antifungal and antibacterial compounds, focusing on works published in the last five years, clearly outlines the directions that have emerged in recent years, and prospects for the development of research in the field of bread preservation and safety.

To summarize the data on the most common microorganisms in bread sourdoughs, we performed the term mapping from OpenAlex text data. Terms were extracted from the title and abstract fields of 1967 documents, containing the term “sourdough” in the abstract. Binary counting was used to determine the frequency of publications mentioning certain species of microorganisms. The term co-occurrence was at least five documents. Direct counting was used to determine the depth of the study. Among the extracted terms, the species names of microorganisms were selected, and the mention number for each species was summed up, taking into account abbreviations. We carried out a direct search for studies in the field of bread sourdoughs in the Scopus, Web of Science, and Pub Med databases from 2020 to 2025 using combinations of the following keywords in search queries: sourdough AND (inhibitory OR antimicrobial OR antifungal OR antagonistic OR antibacterial OR bacteriostatic), as well as antimold/antimold, microbiota, “lactic acid bacteria”, “bread spoilage”, rope, and others. The main research areas were identified for relevant articles where necessary, and a more specific search was conducted when needed, including in the Google Scholar database and in extended time intervals.

This review provides a detailed and thorough summary of the latest research results on the effects of bread starters on improving bread shelf life and safety, highlighting the main directions and new strategies.

The genera of LAB are presented in the review in a modern classification [12].

## 2. Sourdough Microbiota

### 2.1. Mapping Terms Among Sourdough-Related Documents

**Figure 1:**
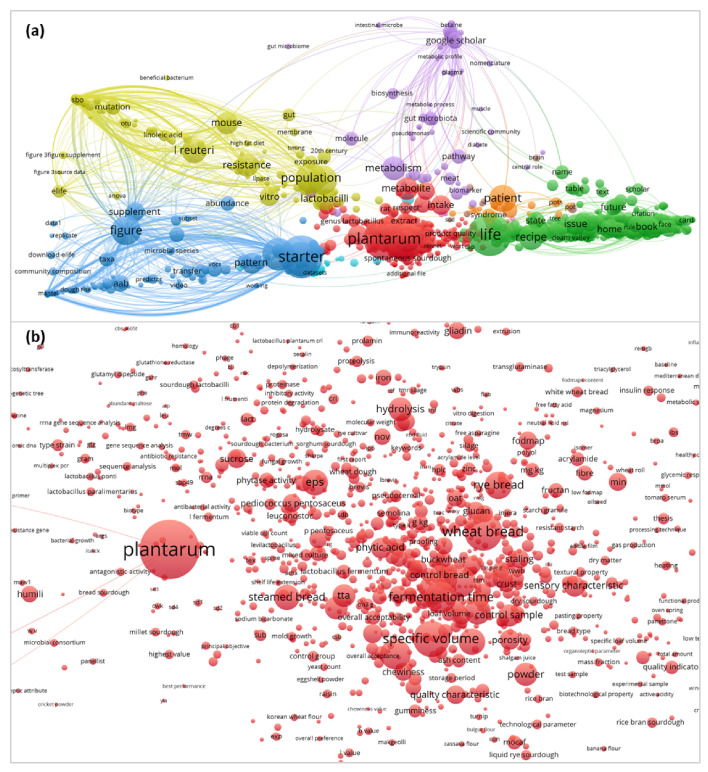
VOSviewer 1.6.20 mapping of terms repeated at least five times in articles with the term “sourdough” in the abstract, based on OpenAlex API text data. (**a**) The group containing the bulk of the species names occupies a central position among other groups of terms; (**b**) *Lactiplantibacillus plantarum* dominates among terms directly related to sourdoughs.

A total of 46,852 terms were extracted from 1967 documents with the word “sourdough” in the abstract. From extracted terms, 2843 were repeated at least five times (binary count relied only on the presence of the term in a publication) (Figure 1a). Terms were automatically placed into several groups by the application. The names of microorganisms were mostly included in the red group (Figure 1b), which contained the largest number of terms and occupied a central position among other groups on the map. In this group, *Lactiplantibacillus plantarum* emerged as the most dominant species.

**Figure 2:**
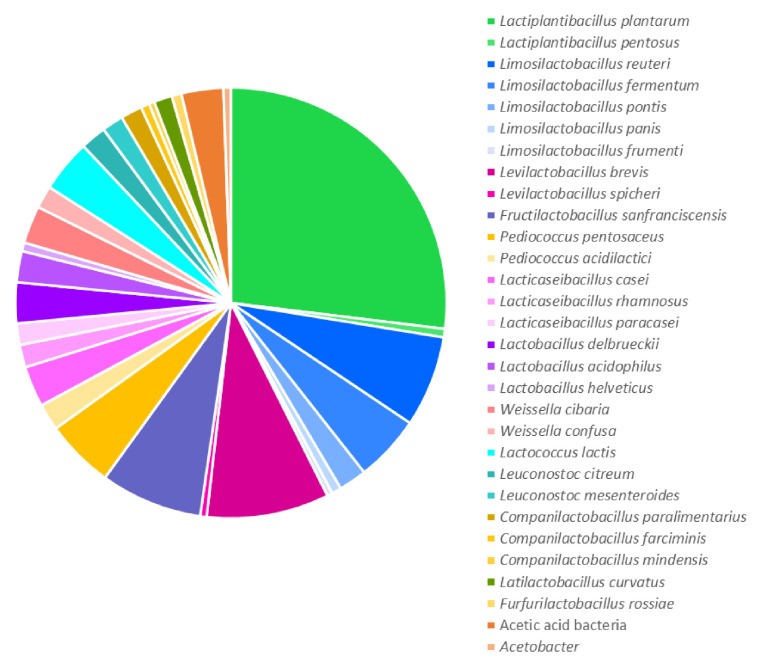
Occurrence of different bacterial species in publications on bread sourdoughs. The diagram is based on VOSviewer mapping (OpenAlex API text data) of terms occurring at least five times in the articles with “sourdough” in the abstract.

Among 1706 most relevant terms, the occurrence of LAB species related to the sourdough microbiota was calculated. Certain species and genera of LAB received more mentions in sourdough literature. Thus, the diagram in Figure 2 represents LAB species repeating in at least five publications from 1971 related to sourdough.

*L. plantarum* occupies a central position in sourdough bread research, accounting for 26.9% of occurrences among species names of microorganisms (Figure 2). This is explained by the fact that this species is abundantly represented in flour of various origins and can dominate its fermentation [13].

LAB of the genus *Limosilactobacillus* are the second most common (15.1% total) after the genus *Lactiplantibacillus*. However, among the species, *Levilactobacillus brevis* (9.2%) and *Fructilactobacillus sanfranciscensis* (7.7%) are slightly more represented in studies compared to *Limosilactobacillus reuteri* (6.8%) and *Limosilactobacillus fermentum* (5.0%). *Pediococcus pentosaceus* also reaches a significant number of mentions (5.2%). The genera of LAB are arranged in the following descending order of frequency of their species occurrence in publications: *Lactiplantibacillus*, *Limosilactobacillus*, *Levilactobacillus*, *Fructilactobacillus*, *Pediococcus*, *Lacticaseibacillus*, *Lactobacillus*, *Weissella*, *Lactococcus*, *Leuconostoc*, *Companilactobacillus*, *Latilactobacillus*, and *Enterococcus*. Studies of the dynamics of microbial successions during sourdough maturation [13] show that microorganisms such as Enterococcus, *Lactococcus*, and *Leuconostoc* are characteristic of the first phase, and *Lactobacillus*, *Pediococcus*, and *Weissella* are characteristic of the second. In the third phase, the microbiota of bread sourdough is dominated by individual species of *Lactobacillus* sensu lato, previously grouped into the genus *Lactobacillus* but transferred to the present time into several new genera [12].

According to the term mapping, the genus *Limosilactobacillus* included the largest number of species, mentioned in at least five publications. In turn, only four genera were represented by a single species. These species were *F. sanfranciscensis*, *Lactococcus lactis*, *Latilactobacillus curvatus*, and *Furfurilactobacillus rossiae*.

At the same time, some microorganisms were mentioned and studied in more detail, as determined by the total term count. Among them are *Limosilactobacillus reuteri* (the occurrence is 12.2% compared to 6.8% in the binary count), acetic acid bacteria (6.8% and 3.1%, respectively), and *Lacticaseibacillus paracasei* (3.0% and 1.6%, respectively). This may indicate some special functions of these microorganisms in bread sourdoughs.

**Figure 3:**
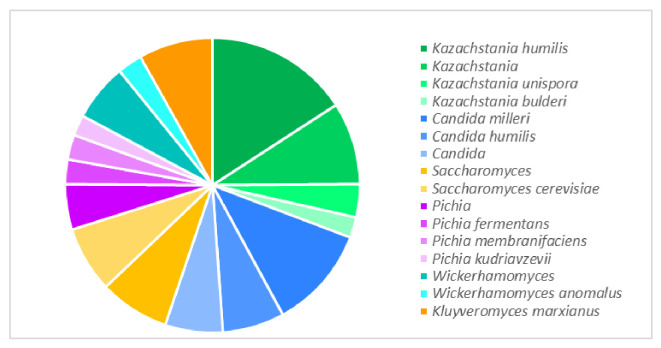
Sourdough fungal microbiota based on VOSviewer mapping (OpenAlex API text data) of terms occurring at least in five articles with the term “sourdough” in the abstract.

In the sourdough yeast microbiota, the most frequently reported genera were *Kazachstania* (30.8%), *Candida* (24.4%), *Saccharomyces* (14.9%), *Pichia* (12.7%), *Wickerhamomyces* (9.0%), and *Kluyveromyces* (8.1%) (Figure 3). The yeast species *Kazachstania humilis* (formerly *Candida humilis*) and *Candida milleri* were the most frequent in sourdough publications.

### 2.2. Types of Sourdoughs

Various researchers have described sourdough microbiota according to the type of production method and fermented flour. There are three main types of sourdoughs, differing in the production method [13,14]. Type I sourdough is an artisanal spontaneous sourdough obtained by back-slopping—renewal using a fresh mixture of flour and water repeated 5–13 or more times. According to De Bondt et al.’s scoping review [15], about 18% of articles on sourdough are devoted to Type I sourdoughs. Type II sourdoughs refer to industrial liquid sourdoughs and are based on pure cultures of LAB added to yeast in a ratio of 100:1 [14]. This type is most often described in the literature because it includes known strains of microorganisms with easily standardized technology for obtaining sourdough and dough. Type III sourdough is an industrial dry sourdough. Only about 8% of publications are devoted to dry sourdoughs [15]. Sometimes, Type IV sourdough is distinguished, which is a combination of Type I and Type II.

#### 2.2.1. Type I Sourdough

Type I sourdough is usually a mixture of yeasts and mesophilic LAB, typical for a particular flour type. The formation of microbiota in spontaneous sourdoughs occurs gradually. In the first stage, cereal microbiota microorganisms such as *Enterobacter*, *Acinetobacter*, *Pseudomonas*, and *Sphingomonas* are found in large quantities [13]. *Klebsiella*, *Serratia*, *Erwinia*, *Clostridium*, and *Staphylococcus* were also identified as dominant genera in the first stage [16]. From day 2 to day 5, *Enterococcus*, *Lactococcus*, and *Pediococcus* cocci predominate, and *Weissella* spp. and *Lactobacillus* (in particular, homofermentative *Lactobacillus delbrueckii* subsp. *lactis*) are also detected [13]. However, the transition to the second stage can be observed much earlier. Thus, De Angelis et al. [16] showed a high number of *Lactococcus*, *Enterococcus*, and *Weissella* already after 8 h. A more rapid change in microbial communities is more characteristic of whole-grain dough. Thus, Boreczek et al. [17] showed that the total number of *Enterococcus*, *Lactococcus*, and *Leuconostoc* did not exceed 2% after 24 h, when studied the microbiota of spontaneous sourdoughs from whole grain flour (wheat, rye, and spelt) during the period of community formation (24–72 h). The reduction in the number of acid-forming cocci is probably due to a rapid increase in acidity, which contributes to sourdough stabilization and dominance of resistant lacticobacilli species. In the studies of Boreczek et al. [17], *Weissella* was the most abundant genus after 24 h of rye sourdough fermentation, and its abundance decreased thereafter in all sourdoughs. In the Oshiro et al. [18] studies, *Weissella* spp. dominated in environments with a more neutral pH, while *L. brevis* was attached to lower pH values.

The relative abundance of *Enterococcus*, *Lactococcus*, and *Leuconostocaceae* in the sourdough varied across geographical zones and wheat varieties; in contrast, rod-shaped LAB did not dominate the microbial communities of the untreated raw materials [19]. Maintaining the sourdough for several days and increasing its acidity leads to the dominance of certain species of lactobacilli that are well adapted to the sourdough ecosystem, such as *L. plantarum* and *L. fermentum* [13]. Interestingly, *Latilactobacillus curvatus*, *L. brevis*, and *Lactiplantibacillus* sp., which dominate in rye sourdoughs during the first three days, were replaced after one month in the stiff sourdough with *F. sanfranciscensis* and *Companilactobacillus* sp., and in the liquid sourdough with *Limosilactobacillus pontis* [20].

A shift in LAB from the genera *Weissella* and *Lactococcus* to *Pediococcus* and *Leuconostoc*, and at later stages to *Lactobacillus*, is characteristic of the spontaneous fermentation of plant materials [21,22,23]. The reduction in cocci and active proliferation of lactobacilli coincide with a decrease in substrate pH, since they are usually acid resistant.

Dissimilar results were obtained by Baev et al. [24]. They showed high *Weissella* contents in whole grain wheat sourdoughs (16.98–42.84%). In addition, mature sourdoughs included 3.24–20.05% *Pseudomonadota* (former *Proteobacteria*), as well as significant amounts of *Cyanobacteria* in some sourdoughs. Also, in one of the mature industrial sourdoughs studied by Debonne et al. [25], *Weissella* was the dominant genus (73.8%, of which 62.2% was *Weissella confusa*). *Leuconostoc citreum* in this sourdough was 3.6%, and 14.5% were other bacteria. Most likely, these features are related to the sourdough production technology, but the studies mentioned do not provide information on the methods for obtaining mature sourdoughs.

The production technology has a significant impact on the sourdough microbiota. The time required for sourdough maturation varies depending on the substrate used. For example, Weckx et al. [26] showed that reverse mowing of rye sourdough resulted in a complete change in the main genera and species of LAB from *Lactococcus*, *Leuconostoc*, and *Weissella* via *L. curvatus* and *P. pentosaceus* to *L. fermentum* and *L. plantarum* in just four days. There is evidence of a relative abundance of *Leuconostoc* after 10 days of back-slopping [16]. The studies of De Angelis et al. [16] also demonstrated the dominance of *P. pentosaceus* and a high relative abundance of *Weissella* species in both soft wheat (42–72%) and durum wheat (19–35%) sourdoughs. The reason for these discrepancies lies in the different sourdough production technologies. The dough in the studies was fermented for only 6–8 h per day, followed by a resting period (16–18 h at 4 °C), preventing acid-forming cocci’s subsequent replacement by acid-resistant rods.

Specific technological parameters such as temperature, dough yield, and the addition of salt affect the biodiversity and the increase in the number of certain LAB species in the sourdough [27]. Sourdough-associated LAB originate from flour, production environment, and other ingredients, if available [19]. Minervini et al. showed the influence of the propagation environment on the composition of sourdough yeast and LAB microbiotas [28]. At the same time, technological features, home characteristics, and climatic factors explained only 9% and 5% of the variations in the composition of bacterial and fungal communities, respectively [29]. In the studies by Minervini et al. [28], major differences between the microbiota of artisan bakery- or laboratory-propagated sourdoughs were shown for yeasts, which are spore-forming microorganisms that survive well on various work surfaces, and for LAB of the genus *Leuconostoc* forming biofilms due to increased production of extracellular polysaccharides [30,31,32]. Therefore, the environmental microorganisms of spontaneous sourdoughs can be attributed to the flour microorganisms that survive during the production process on utensils and work surfaces, rather than to the externally introduced microbiota. Later, Minervini et al. [33] demonstrated that endophytic LAB of the cereals used for flour production form the basis of sourdough microbiota. The authors showed that *Lactobacillus*, *Streptococcus*, *Enterococcus*, and *Lactococcus* were the main LAB genera in durum wheat epiphytic and endophytic microbiota. The relative abundance of genera depends on plant varieties, phenological stages, environmental factors, and agronomic techniques. The dominance of *L. plantarum* in various sourdoughs (Figure 1) is explained by its association with wheat plant organs throughout the life cycle.

It has been shown that Type I wheat and rye sourdoughs are usually colonized by insect-adapted lactobacilli such as *F. sanfranciscensis*, while Type II sourdoughs usually include vertebrate-adapted lactobacilli of the *L. delbrueckii* and *L. reuteri* groups [34]. In contrast, *F. sanfranciscensis* and *Pediococcus parvulus* were reported to be more abundant in older sourdoughs, while *L. plantarum* was more numerous in younger sourdoughs [20,29]. *F. sanfranciscensis* was also more often identified in private sourdoughs originating from a commercial source, while *L. brevis* prevailed in de novo sourdoughs [29]. The latter showed good growth performance in a separate culture and was well preserved in a pair with competing LAB and yeast, whereas the former species was characterized by low competitive ability and could survive only with the yeast *K. humilis*. It is known that *F. sanfranciscensis* is characterized by the smallest genome size among lactobacilli and a low guanine-cytosine content, which indicates adaptation to a narrow ecological niche [34,35]. The highest density of ribosomal RNA operons confirms the reductive evolution of this species and indicates a high frequency of gene inactivation and elimination [35]. *F. sanfranciscensis* is the most abundant and predominant lactobacilli species in fecal samples of confused flour beetle (*Tribolium confusum*) and, consequently, insect excreta from stored grain products may be a natural reservoir of these bacteria [36]. At the same time, the ability of *F. sanfranciscensis* to synthesize de novo deficient in wheat amino acids facilitates its good growth in a sourdough environment [35].

Landis et al. [29] studied 500 sourdoughs mostly from the United States (429) and found strong patterns of species dominance or co-occurrence. Most sourdoughs were dominated by a single yeast and/or bacterial species, with a median of three LAB and/or acetic acid bacteria (AAB) and one yeast species per sourdough. *Saccharomyces cerevisiae* was the most abundant yeast in the studied sourdoughs, followed by *F. sanfranciscensis* among the bacteria. *L. sanfranciscensis* was also shown to be negatively correlated with *L. plantarum* and *L. brevis*. *L. brevis*, in turn, was the most frequently co-occurring species. Despite the widespread occurrence of *S. cerevisiae* and *F. sanfranciscensis*, these microorganisms tended to be mutually exclusive, while *K. humilis* was more often found together with *F. sanfranciscensis* [14,25,29]. However, the dominance of *S. cerevisiae* among yeasts was also documented for *F. sanfranciscensis*-dominated sourdoughs [25,37]. At the same time, AAB and other LAB were negatively correlated with *F. sanfranciscensis* [37].

Interesting data were obtained by Xing et al. [38], indicating the influence of terrain conditions on the composition of sourdoughs. *Acetobacter* was widespread only in the mountain samples, while *P. pentosaceus* was the dominant strain in the basin sourdough samples.

AAB are now known as constant companions of natural fermentation of sugar-containing substrates by LAB and yeasts [39]. Although AAB rarely dominate due to strictly aerobic metabolism, they influence metabolic pathways and final product properties. They are also a common and characteristic component of spontaneous sourdoughs [29,37,40,41,42]. Landis et al. [29] detected AAB in 147 sourdough samples of 500 with a relative abundance of more than 1%. In another study, AAB were detected in 29.4% of 500 sourdough samples from different continents, with an average relative abundance of 23.3% [41]. Interestingly, all Belgian sourdoughs investigated by Comasio et al. [37] contained AAB. Nevertheless, the absence of a sourdough-specific genomic cluster indicates that AAB are nomadic microorganisms [41].

The presence of AAB in bread sourdoughs has not been systematically investigated, so data on their abundance are incomplete. Several AAB species belonging to *Acetobacter* and *Komagataeibacter* were detected in sourdoughs [37]. Isolated AAB species included *Acetobacter cerevisiae*, *Acetobacter oryzifermentans*, *Acetobacter senegalensis*, *Acetobacter fabarum*, *Acetobacter pasteurianus* subsp. *pasteurianus*, *Acetobacter sicerae*, *Komagataeibacter xylinus*, and *Komagataeibacter sucrofermentans*. In turn, shotgun metagenomic sequencing by Landis et al. [29] showed the presence of *Acetobacter malorum*, *A. pasteurianus*, and *Acetobacter lovaniensis* in sourdoughs. The most common AAB were *Acetobacter malorum*/*cerevisiae* and *Acetobacter oryzoeni*/*oryzifermentans*/*pasteurianus*. The genera *Gluconobacter* and *Komagataeibacter* were also noted.

The functions of AAB and their importance in sourdoughs remain to be fully understood.

#### 2.2.2. Type II Sourdough

In Type II sourdoughs, the dominant microorganisms are LAB, and *S. cerevisiae* is added for leavening [7]. The choice of starter cultures for the most common Type II sourdoughs traditionally used in industry depends on the metabolic traits of technological and functional interest. The use of sourdough makes it possible to influence the sensory and rheological properties of bread, its shelf life, and safety. Sourdough can also improve the nutritional and functional properties of bakery products, enriching them with vitamins, essential amino acids, fatty acids and bioactive peptides, reducing the glycemic index and gluten content, and increasing the bioavailability of minerals [19,43,44].

Important parameters for starter microorganisms are their acidifying capacity and growth characteristics. Other important properties of starter microorganisms include the production of exopolysaccharides and various volatile compounds, as well as proteolytic and antagonistic activities [43,45,46].

Acid-tolerant strains of LAB belonging to the genera Limosilactobacillus and Lactobacillus [7] are commonly used for sourdoughs, such as Lactobacillus amylovorus, Limosilactobacillus panis, L. pontis, and Limosilactobacillus reuteri [14]. F. sanfranciscensis is often used as the sole leavening agent [7].

LAB proteolytic activity is crucial for the degradation of flour proteins for affecting bread texture and the production of bioactive peptides. De Vero et al. [21] revealed significant proteolytic activity of *L. citreum* PRO17 and *P. pentosaceus*, strains OA1 and S3N3, in whole wheat flour dough. Angiotensin I-converting enzyme, for controlling hypertension, and cancer-preventive peptide lunasin are some bioactive compounds of sourdough LAB. As De Vero et al. showed, *L. curvatus* SAL33 and *L. brevis* AM7 significantly increased the concentration of lunasin [21]. In turn, *L. amylovorus* DSM19280, *L. brevis* R2Δ, and *L. reuteri* R29 revealed good results as microorganisms with high antifungal activity, producing a total of 171–589 mg/kg antifungal compounds [47]. The antimicrobial activity of LAB from sourdoughs will be discussed in more detail in the following sections.

Microorganisms originating from sources other than the spontaneously produced microbiota of dough and cereals are often considered promising for Type II sourdoughs. Thus, Comasio et al. [48] studied the adaptation of LAB isolates from other sources to a cereal matrix and showed the promise of using *L. fermentum* from cocoa mass and *Latilactobacillus sakei* from fermented sausage in wheat sourdoughs. Zhang et al. [49] found high cell counts of *Lentilactobacillus buchneri* from African cereal products and *Lentilactobacillus diolivorans* from maize silage in wheat, rye, and buckwheat sourdoughs. The microorganisms also produced acetic and propionic acids, inhibiting the growth of *Aspergillus clavatus*, *Cladosporium* spp., and *Mortierella* spp. Another study [50] demonstrated the effectiveness of *L. plantarum* from pickles against the bakery spoilage fungi *Penicillium citrinum*, *Aspergillus flavus*, *Aspergillus fumigatus*, *Fusarium graminearum*, *Aspergillus niger*, and *Aspergillus ochraceus*. While Muhialdin et al. [51] showed shelf life extension of bread inoculated with *A. niger* and *Aspergillus oryzae* using LAB (*L. fermentum*, *P. pentosaceus*, *L. pentosus*, and *L. paracasei*) isolated from Malaysian fruits and fermented products. *L. plantarum* ZZUA493 derived from alfalfa inhibited the growth of *A. niger*, *A. oryzae*, *Trichoderma longibrachiatum*, *A. flavus*, and *F. graminearum* in Chinese steamed buns through the production of lactic, acetic, and phenyllactic acids [52]. At the same time, dairy (kefir grain-derived) *P. pentosaceus* SP2 and *L. paracasei* SP5 enhanced the resistance of baked bread to both fungal and rope spoilage [53,54].

AAB have largely escaped research attention due to the difficulties of maintaining them in pure culture. However, the detection of significant amounts of AAB in spontaneous fermentations of various substrates with LAB and yeast indicates the need for a more thorough investigation of the contribution of AAB to co-fermentation and the properties of the resulting products. To date, few studies have investigated the contribution of AAB to sourdough fermentation. However, contributions of AAB to dough acidification (by 18.5% compared to yeast and LAB), volatile aroma profile, amino acid biosynthesis, and dough properties (rise, viscosity, and elasticity) have been reported [29,37,41,55,56].

Research on the contribution of AAB to bread sourdoughs has only begun in recent years, but several studies have already shown promise for their use. Considering the production of acetic acid, the antimicrobial properties of different types of vinegar [57,58], the information on the novel antimicrobial properties of AAB in combination with LAB [59,60,61], and the involvement of AAB in the biodetoxification of mycotoxins [62,63,64], the influence of AAB on the shelf life and safety of bread should be expected.

#### 2.2.3. Type III Sourdough

Desiccation-resistant LAB, such as *P. pentosaceus*, *L. plantarum*, and *L. brevis*, usually predominate in Type III sourdoughs [7,65,66]. However, the survival of microorganisms in such starters depends on the drying process. Therefore, Type III sourdoughs are often added to doughs only to improve the taste of bread.

Nevertheless, the convenience of dry sourdoughs encourages researchers to develop efficient technologies for their preparation and use. The research on dry sourdoughs is still in the developmental stage. However, the studies by Lafuente et al. [66] showed the preservation of bread prepared with dry sourdough fermented with *P. pentosaceus* TI6, which was experimentally infected with *A. flavus* and *Penicillium verrucosum*. In turn, Teixeira et al. [67] showed an extension of the shelf life of bread (especially from whole grain flour) using 10% (*w*/*w*) Type III sourdough with starter cultures of *F. sanfranciscensis* and *L. plantarum*. The water-soluble sourdough extract inhibited *P. roqueforti*, *P. chrysogenum* and *A. niger*. The best effect (shelf life of 10 days) was shown by sourdough from a mixture of wheat and flaxseed flour fermented with *F. sanfranciscensis*. In other studies [68], probiotic cultures of *Lactobacillus acidophilus*, *Lacticaseibacillus casei*, *Bifidobacterium* spp., and *Bacillus coagulans* were used in combination with a spray-dried sourdough fermented with LAB and propionibacteria. Its application of 1% (*w*/*w*) extended the shelf life of pizza and focaccia bases by an additional 10 days.

#### 2.2.4. Type IV Sourdough

Type IV sourdough is a mixture of traditional Type I and Type II sourdoughs, where the starter cultures are added to a mature spontaneous sourdough [69]. Type IV sourdough can also be dried [70]. Another option for preparing a Type IV sourdough is adding starter based on pure LAB cultures to the dough with subsequent daily renewal [71].

## 3. The Effect of Sourdough LAB on the Shelf Life of Bread

### 3.1. Antifungal Compounds

Sourdough LAB have been shown to inhibit the following mold fungi: *A. fumigatus*, *A. niger*, *A. oryzae*, *Aspergillus versicolor*, *Aspergillus japonicus*, *A. clavatus*, *Aspergillus chevalieri*, *Aspergillus montevidensis*, *P. roqueforti*, *P. chrysogenum*, *Penicillium expansum*, *Penicillium crustosum*, *Penicillium olsonii*, *Penicillium polonicum*, *P. corylophilum*, *F. oxysporum*, *F. moniliforme*, *F. culmorum*, *Mucor* spp., *Rhizopus* spp., *Cladosporium* spp., *Neurospora* spp., *Mortierella* spp., and others [2,72,73,74,75,76].

Various researchers have shown that the addition of 20–30% (*w*/*w*) LAB-based sourdough to the dough extends the shelf life by 1 to 25 days, depending on the selected microbial strains [7,77,78]. Along with the shelf life extension of bread baked using sourdough, an improvement in texture and other consumer and production-relevant attributes is often noted [43]. Sensory properties are assessed in various studies in the range from slightly below control to above control, which is associated with an increase in the diversity and content of volatile compounds and individual consumer preferences [78,79].

#### 3.1.1. Organic Acids

Many researchers note that the main mechanism of LAB that exerts an antifungal effect is the production of organic acids. A mixture of lactic, phenyllactic, and acetic acids is usually reported [47,65,73,76,78]. For example, aqueous extracts of wheat flour fermented with *L. acidophilus* and *L. casei* produced 1–2% (*w*/*v*) lactic acid, which reduced the growth rate of the molds *P. crysogenum* and *P. corylophilum* [73]. In another study, cell-free supernatants of several LAB (*W. cibaria*, *L. plantarum* subsp. *plantarum*, *L. pseudomesenteroides*, *F. sanfranciscensis*, *L. brevis*, and *L. pentosus*) contained 4.33–8.41 g L^−1^ lactic acid that inhibited *A. flavus*, *A. niger*, and *P. expansum* [80]. Other researchers estimated an increase in lactic and acetic acid concentrations in sourdough bread with *P. pentosaceus* и *S. cerevisiae* by 4.5 and 1.6 times, respectively, compared to the control [81]. In the studies of Wu et al. [76], *L. plantarum* LWQ17 produced 60 times more phenyllactic acid than *P. pentosaceus* LWQ1, which provided better suppression of *A. niger* and *P. polonicum*. In turn, *L. plantarum* P10 inhibited spore and mycelial growth of *A. niger* in Chinese steamed bread [82], while panettone with *L. fermentum* IAL 4541 contained 5–10.4 mmol/kg phenyllactic acid, 1.17–8.85 mmol/kg acetic acid, and 3.5–4.3 mmol/kg propionic acid [83].

Some researchers use standard unmodified de Mann, Rogosa, and Sharpe (MRS) medium in in vitro studies of LAB antagonism against molds [84,85], which may contribute to obtaining overestimated results regarding the antifungal activity of the isolated strains. It has been shown that sodium acetate of the MRS medium contributes greatly to the antifungal effect [59,86,87]. Studies by Stiles et al. [87] revealed an inhibitory effect of sodium acetate on the growth of 33 out of 42 mold strains from the genera *Fusarium*, *Penicillium*, *Aspergillus*, and *Rhizopus*. The combination of *L. rhamnosus* VT1 and sodium acetate exerted a synergistic inhibitory effect against 39 mold strains. Fraberger et al. [88] suggested that the MRS medium is a good stimulator of the production of antifungal compounds. In our work (unpublished data), the exclusion of sodium acetate from the MRS medium abolished the antagonistic activity of most LAB that inhibited fungi on the standard medium.

The effect of sodium acetate can be explained by the displacement of weak acids from their salts by stronger acids. It is known that the strength of organic acids depends on the length of their hydrocarbon radical, decreasing with its elongation. However, electron-withdrawing substituents enhance the acidic properties of organic acids [89]. This is why lactic acid is more acidic than acetic acid, although its radical is longer. The pKa value of acetic acid is 4.76, while the pKa value of lactic acid is 3.86, and strong acids have pKa values less than zero [90]. These data indicate that the production of lactic acid in a medium containing sodium acetate will lead to the accumulation of undissociated acetic acid. In our studies (unpublished data), gas chromatography–mass spectrometry of homofermentative LAB volatiles in MRS did not reveal lactic acid but indicated the accumulation of acetic acid. Anyway, high production of organic acids helps to suppress the growth of fungal microorganisms, so the selection of active acid formers on a medium with sodium acetate can also be effective, which was confirmed by extended storage of bread for a week in the experiments of Bartkiene et al. [91] without intentional contamination.

Debonne et al. [25] demonstrated the importance of undissociated acids in improving the shelf life of bread. In their studies, air-packed bread prepared with 30% (*w*/*v*) sourdough, fermented by *L. sanfranciscensis* and *S. cerevisiae*, did not deteriorate over the entire 7-week observation period. Sourdough bread contained 36 mmol undissociated lactic acid and 220 mmol undissociated acetic acid per liter of the aqueous phase. It was also shown that the main effect depended on undissociated acetic acid, and lactic acid was insignificant in inhibiting fungal growth, only contributing to the overall acidification. A concentration of 150–200 mmol undissociated acetic acid per liter of the aqueous phase increased the shelf life of the bread. It has also been shown that different fungi are sensitive to the effects of acetic acid to varying degrees. Thus, the growth of *A. niger* was completely inhibited in chemically acidified bread at an acetic acid concentration above 33 mmol/kg dough (which corresponds to 165 mmol undissociated acetic acid in one liter of the aqueous phase of bread), while the growth of *Penicillium paneum* was inhibited only at a concentration above 100 mmol kg^−1^ dough [92]. In another study Debonne et al. [93] assessed the effect of undissociated acetic acid and phenyllactic acid on the growth of *P. paneum* and *A. niger* fungi and the shelf life of semi-baked bread. The minimum inhibitory concentration (MIC) of undissociated acetic acid in bread was 110–169 mM, which was sufficient to prevent mold growth for 45 days. The MIC of phenyllactic acid was 39–84 mmol L^−1^ aqueous phase. It was concluded that the naturally occurring phenyllactic acid in sourdough bread is not sufficient to extend the shelf life of bread and that acetic acid is the most important antifungal agent in sourdough bread. The MIC value for phenyllactic acid is consistent with the previously reported MIC value of 7.5 mg mL^−1^ (45 mmol L^−1^) against *A. fumigatus* and *P. roqueforti* by Ström et al. [94]. In the study by Lavermicocca et al. [95] MIC of phenyllactic acid was 8.3 mg/disk against *P. roqueforti* IBT18687 and 2.5 mg/disk against *Endomyces fibuliger*. Unfortunately, MIC data of phenyllactic acid against other fungi are not presented in the work, despite the information on the suppression of a wide range of mold fungi (*Eurotium repens*, *Eurotium rubrum*, *P. corylophilum*, *P. roqueforti*, *P. expansum*, *Endomyces fibuliger*, *A. niger*, *A. flavus*, *Monilia sitophila*, and *F. graminearum*) by 10-fold-concentrated *L. plantarum* 21B culture filtrate in wheat flour hydrolysate. Maximum antifungal activity was found in the ethyl acetate extract of the culture filtrate, which contained the highest concentration of phenyllactic acid. However, the authors reported no synergistic effect between the MIC of phenyllactic acid and non-inhibitory concentrations of p-hydroxyphenyllactic and palmitic acids.

Formic acid at 24.7 mM, formed during fermentation of wheat germ by LAB *L. plantarum* LB1 and *F. rossiae* LB5, showed high antifungal activity and acted in a mixture with four peptides [96]. Propionic acid is determined among LAB metabolites much less frequently than lactic and acetic acids.

However, a stronger inhibitory effect of propionic acid (MIC from 8 to 20 mmol/liter) was shown, compared to acetic acid (MIC from 23 to 72 mmol/liter) against *A. niger*, *P. corylophilum*, and *E. repens* [97]. Acetate and propionate produced by *Lentilactobacillus buchneri* FUA 3252 and *Lentilactobacillus diolivorans* DSM14421 inhibited bread spoilage by *A. clavatus*, *Cladosporium* spp., and *Mortierella* spp. in the study by Zhang et al. [49]. At the same time, increasing the proportion of sourdough from 10% to 20% (*w*/*w*) also inhibited the growth of baker’s yeast *S. cerevisiae* in dough. Therefore, the dosage of antifungal compounds should be adjusted to take into account the effect not only on molds but also on the yeast used in bread production.

The list of antifungal organic acids produced by LAB in soudfough includes lactic, acetic, formic, propanoic, hydrocinnamic acid, phenyllactic and hydroxyphenyllactic, butyric, n-valeric, 2-hydroxyisocaproic, capric, ρ-coumaric, (E)-2-methylcinnamic, azelaic, gallic, caffeic, ferulic, hydrocaffeic, hydroferulic, syringic, sinapic, monohydroxy octadecenoic, phloretic, salicylic, vanillic, and other acids [2,6,7,14,47,98,99,100] (Table 1).

However, despite the wide range of LAB antifungal compounds, their detected concentrations are often lower than the known MICs [47]. At the same time, combinations of LAB organic acids can have a synergistic antifungal effect. However, the contribution of different acids to the overall synergistic effect is not the same. Thus, Corsetti et al. [101] showed that *F. sanfranciscensis* CB1 suppressed *A. niger*, *F. graminearum*, *P. expansum*, and *M. sitophila* to varying degrees. The antifungal effect was synergistic between acetic, caproic, formic, propionic, butyric, and n-valeric acids. These acids did not inhibit the growth of the studied fungus *F. graminearum* individually in the produced concentrations, but at a dose corresponding to the sum of all acids (16 mM), each acid gave inhibition halos. The inhibition zones of *F. graminearum* were maximum when using all six acids. However, the decisive role in the manifestation of the antifungal effect was played by caproic acid, followed by acetic acid. The input of other acids in increasing antifungal activity was weak.

The water-soluble extract of *L. bulgaricus* contained phenyllactic and sinapic acids [99], while *L. plantarum* CECT 749 produced several phenolic acids, namely gallic, caffeic, chlorogenic (an ester of caffeic and quinic acids), and syringic acids. Chlorogenic acid was also detected in wheat-whey dough fermented by *L. bulgaricus* CECT 4005. Both species produced vanillic acid in wheat-whey dough. Water-soluble extracts from wheat dough fermented by *L. plantarum* inhibited *P. expansum*, *P. roqueforti*, *P. camemberti*, *F. moniliformis*, *F. graminearum*, *F. verticillioides*, *A. niger*, and *A. parasiticus*. *L. bulgaricus* extracts were active only against *Fusarium* species and *P. expansum*. However, the MICs of the organic acids were lower than those of *L. plantarum*. *A. flavus* was also inhibited by both LAB when whey powder was added to the dough. Bread contaminated with P. expansum baked with *L. bulgaricus* had a shelf life one day longer than that baked with *L. plantarum*. These results suggest that phenyllactic and sinapic acids were more effective in inhibiting *P. expansum*, while the other identified phenolic acids contained in the *L. plantarum* extract were better inhibitors of *P. camemberti*, *P. roqueforti*, and *A. parasiticus*.

Often, the most significant factor in inhibiting the growth of fungal microorganisms is the production of organic acids. Thus, neutralization of LAB cell-free supernatants led to a reduction in the zones of inhibition of mold fungi *P. chrysogenum*, *F. graminearum*, for *Rhizopus stolonifer* and *Aspergillus nidulans* by 67.0–77.4% [106]. The use of neutralization of cell-free supernatants to exclude the effect of medium pH [72] allows the selection of microorganisms with other mechanisms of action. Axel et al. [47] also showed that chemical acidification with a mixture of lactic and acetic acids does not allow achieving the activity level of sourdough fermented by *L. amilovorus* DSM19280, *L. brevis* R2Δ, and *L. reuteri* R29.

At the same time, medium-chain fatty acids may affect fungal cells not by lowering the pH, but mainly due to the fatty acid radical, which, probably having surface activity, has a direct damaging effect on cell membranes. Thus, sodium decanoate had the lowest MIC among all the studied compounds of *L. amylovorous* DSM 19280, which also included carboxylic acids, cyclic-dipetides, and nucleosides [103], and Sadeghi et al. [102] showed the suppression of *A. niger* by the active fraction of *L. reuteri* CFS containing n-decanoic, 3-hydroxydecanoic, and 3-hydroxydodecanoic acids.

Thus, LAB associated with bread sourdoughs were found to produce several organic acids, primarily saturated aliphatic acids with a short carbon chain, and a large set of acids containing hydroxy and phenolic substituents, including those with an unsaturated carbon skeleton. Benzoic acids were also noted.

Suppression of bread spoilage fungi by organic acids strongly depends on the level of their LAB production. As studies show, the levels of LAB organic acid production vary significantly. Some studies have shown the leading contribution to the suppression of mold fungi by such acids as acetic, 3-phenyllactic, lactic, caproic, propionic, and formic. Other acids were more often produced in concentrations below MICs and had only an auxiliary effect.

#### 3.1.2. Other Compounds

Except for organic acids, LAB antifungal compounds also include hydrogen peroxide, reuterin, diacetyl, peptides and cyclic dipeptides, proteinaceous compounds, and phenolic compounds [6,7,14,47,98,100,104]. The main effect of LAB is synergistic between pH and other antifungal metabolites [7,14]. Bacteriocins produced by LAB can also affect fungi. Thus, Ai et al. [107] showed that nisin of *L. lactis* was retained during baking and reduced fungal contamination. Other researchers have also demonstrated the antifungal activity of nisin [108,109]. *L. plantarum* is known to produce a two-peptide bacteriocin, plantaricin [110], which may be effective against Gram-positive bacteria of the genus Bacillus [111,112] and molds of the genera *Aspergillus*, *Fusarium*, *Mucor*, and *Penicillium* [113].

Reuterin (3-hydroxypropionaldehyde) is specific to *L. reuteri* and likely the reason for the more extensive study of this microorganism (Section 2.1, Figure 2). However, in the studies of Schmidt et al. [107], the antifungal activity of reuterin from *L. reuteri* R29 was not transferred to the bread system. On the other hand, the increased accumulation of phenyllactic acid by this microorganism affected the shelf life of bread. Another factor explaining the extensive study of *L. reuteri* in bread making is the production of the exopolysaccharide reuteran, which improves bread volume and texture [114].

Diacetyl was also not detected in sourdoughs at levels above the MIC [77].

Studies have shown [115] that short-chain organic acids produced by LAB, such as formic, acetic, propionic, oxalic, and lactic acids, exert a synergistic effect with H_2_O_2_ accumulated by LAB due to their lack of catalase [116].

The accumulation of acids in sourdough is often noted as a factor acting in concert with protein and peptide compounds. Thus, Illueca et al. [9] showed increased concentrations of lactic and phenolic acids in breads fermented with *L. plantarum* with simultaneous hydrolysis of proteins (50 kDa). In turn, Hernandez-Figueroa et al. [117] identified protein fractions in wheat sourdough fermented with *L. plantarum* NRRL B-4496, in addition to lactic and acetic acids, with high antifungal activity against *P. chrysogenum* and *P. corylophilum*. The molecular weight of the antagonistically active fraction was greater than 30 kDa. At the same time, neutralization of poolish-type sourdough aqueous extract decreased antifungal activity from about 91% to 3.25–9.65%. After hydrolysis of the extract with proteinase K, the suppression was less than 1%. Thus, the contribution of protein fractions to the suppression of *P. corylophilum* and *P. chrysogenum* growth was 2.58% and 9.01%, respectively. It has been shown [118] that *L. plantarum*-based sourdough is a good natural alternative to sodium propionate as an antifungal agent in bread. Some protein-like compounds of *L. fermentum* Te007 and *P. pentosaceus* Te010 prevented *Aspergillus* spoilage of bread [51], while compounds less than 10 kDa were responsible for the antifungal activity of *L. mesenteroides* DU15 and three *L. plantarum* strains at pH 3 [119].

In another study [120], antifungal metabolites of *P. pentosaceus* from wheat sourdough included a fatty acid ester, a hydroxylated fatty acid ester, 3-isobutyl 2,5 piperazinedione, and a cyclic dipeptide. It was previously shown [94] that *L. plantarum* MiLAB 393 can produce antifungal cyclic dipeptides (diketopiperazines) cyclo(L-Phe–L-Pro) and cyclo(L-Phe–trans-4-OH-L-Pro). MIC for cyclo(L-Phe–L-Pro) against *A. fumigatus* and *P. roqueforti* was 20 mg mL^−1^. This cyclic dipeptide revealed a weak synergistic effect in combination with phenyllactic acid (MIC 7.5 mg mL^−1^), with both compounds at concentrations below their MICs (10 mg mL^−1^ and 5 mg mL^−1^, respectively). In turn, Dal Bello et al. [121] isolated two *L. plantarum* FST1.7 antifungal dipeptides: cyclo (L-Leu-L-Pro) and cyclo (L-Phe-L-Pro). These peptides were active against bread spoilage *Fusarium culmorum* and *F. graminearum*. Later, Ryan et al. [103] identified five antifungal cyclic dipeptides in *L. amylovorous* DSM 19280 broth that were effective against *A. fumigatus* at concentrations >25–50 mg mL^−1^. All compounds contained proline as one of the amino acids. In addition to cyclo (L-Leu-L-Pro), the cyclic dipeptides also included cyclo(L-His-L-Pro), cyclo (L-Pro-L-Pro), cyclo (L-Met-L-Pro), and cyclo (L-Tyr-L-Pro). The study by Ryan et al. [103] simultaneously demonstrated antifungal activity of high doses (>200 mg mL−1) of cytidine nucleosides and 2′-Deoxycytidine of *L. amylovorous* DSM 19280 against *A. fumigatus*. In earlier work, Ryan et al. [122] showed that cyclic dipeptides are not LAB metabolic products, since the amount of diketopiperazines in acidified dough did not differ from that in dough obtained using sourdough. Axel et al. [98] also showed that *L. brevis* R2Δ also produces cyclic dipeptides such as cyclo(Leu-Pro), cyclo(ProPro), and cyclo(Phe-Pro).

Nionelli et al. [123] detected nine antifungal peptides with a molecular weight of about 1.1–1.2 kDa and 10–17 amino acid residues during the hydrolysis of bread residues by *L. brevis* AM7. All these peptides were encrypted into the sequences of wheat proteins. Previously, Coda et al. [124] also detected nine antifungal peptides in a water-soluble extract obtained from dough fermented by *L. plantarum* 1A7 (S1A7) and effective against *P. roqueforti*. The identified peptides with a molecular weight from 1.5 to 5.6 kDa were encrypted into sequences of *Oryza sativa* proteins and contained 14–53 amino acid residues. The MICs of identified peptides varied from 2.5 to 11.2 mg mL^−1^.

It is known that endogenous wheat proteinases have an optimum pH of 3.0–4.0 [125]. Therefore, acidification of dough to the optimum value by LAB promotes activation of endogenous proteinases and hydrolysis of wheat proteins with the formation of biologically active peptides. The wheat gluten is a complex mixture of hundreds of proteins, the main ones being gliadin and glutenin [126]. Garofalo et al. [77] found several peptides related to wheat α-gliadin in the active fractions of dough extracts obtained using *F. rossiae* LD108. *L. rossiae* LD108 and *Lactobacillus paralimentarius* PB127 exhibited antagonism against the bread spoilage fungi *A. japonicus*, *E. repens*, and *Penicillium roseopurpureum*. At the same time, the detected acetic acid, phenyllactic acid, and diacetyl were in concentrations below MICs. In the studies of Rizzello et al. [96] in the fermentation of wheat germs, antifungal peptides were found in various proteins (formin-like protein 4, homeobox-leucine zipper protein HOX2, expansin-B4, and probable cation transporter HKT6) with sequences from 7 to 52 amino acids. The MICs of defined peptides ranged from 2.5 to 15.2 mg mL^−1^.

Considering the above information, it can be assumed that the absence of proteins capable of hydrolyzing into antifungal peptides may be one of the possible reasons for the accelerated spoilage of gluten-free bread [2].

Thus, LAB produce a whole list of antifungal compounds, mainly a wide range of organic acids, which can either have a direct damaging effect in an undissociated state inside the cell or damage cell membranes, or activate flour proteinases by lowering the pH with subsequent hydrolysis of its proteins and the production of antifungal peptides.

At the same time, there are also results showing the lack of effectiveness of 10% (*w*/*w*) sourdough fermented with *F. sanfranciscensis* in extending the shelf life of bread contaminated with *A. clavatus* and *P. roqueforti* [127]. Only a decrease in the aW value to 0.92 and the addition of propionate contributed to an increase in the shelf life of contaminated bread. There is also evidence that the visual absence of mold growth is not always accompanied by complete suppression and can simultaneously increase the production of mycotoxins [128]. The research results show the need for careful selection of starter microorganisms to receive positive results and control the level of mycotoxins in the environment.

#### 3.1.3. Mycotoxin Removal

Despite the reduction in mycotoxin levels during bread baking, their concentrations may still exceed the maximum permissible levels at high contamination [128,129]. At high bread consumption, mycotoxin exposure in adults and children may exceed the guideline values [130]. In these cases, the use of sourdough is a good option to improve the safety of contaminated flour. Several studies have shown the possibility of reducing mycotoxin levels by sourdough LAB [9,128,129,131]. For example, Cao et al. [132] showed that *L. plantarum* AR524 inhibited the growth of *F. graminearum* by 60.19% compared to the control, while removing up to 40.9% of deoxynivalenol (DON). Mycotoxin removal occurred primarily through cell wall binding. In the studies of Badji et al. [133], in vitro removal of aflatoxin B1 and ochratoxin A was reported using both viable and nonviable LAB cells of *L. lactis* ssp. *lactis*, *L. paracasei* ssp. *paracasei*, *Enterococcus faecium*, and *Enterococcus durans*. The mycotoxin removal efficiency was strain specific, with aflatoxin B1 being better bound by nonviable cells. In another study [9], *L. reuteri* was also effective in aflatoxin removal.

The duration of fermentation has been shown to affect the removal of mycotoxins. After 48 h, the level of DON in the sourdough was reduced by 44–69% in highly contaminated grain [134]. At the same time, toxins such as 15-acetyldeoxynivalenol, alternariol, deoxynivalenol-3-glucoside, H-2, and HT-2 were completely removed, while the reduction in enniatin depended on its form.

In the investigation of Lafuente et al. [66], a high effect in reducing aflatoxin content in experimentally contaminated bread was shown by dry sourdough fermented with *P. pentosaceus* TI6. Although the main mechanism of mycotoxin removal by LAB is cell wall absorption, their biotransformation into non-toxic by-products is also possible [100,128]. At the same time, since the mycotoxin content is higher in the outer grain layers, bran breads may contain increased amounts of mycotoxins [135,136]. The studies by Vidal et al. [135] did not provide a clear answer regarding the effect of sourdough on the mycotoxin levels in bran breads. In some cases, an increase in toxin levels was noted. Therefore, further research in this area is needed.

### 3.2. Antibacterial Activity

Rope disease is caused by amylase-producing spore-forming bacteria belonging to *Bacillus subtilis* and other closely related genera and species that survive in bread during baking and degrade bread polysaccharides to form mucus, for example, *Bacillus amyloliquefaciens*, *B. licheniformis*, the *B. cereus* group, *B. pumilus*, *B. sonorensis*, *Cytobacillus firmus*, *Niallia circulans*, *Paenibacillus* spp., *Lysinibacillus* spp., and *Priestia megaterium* [10,137]. Due to consumer preference for preservative-free products, a re-emergence of the rope disease problem is expected. Global warming exacerbates this problem due to the abundant development of temperature and desiccation-resistant endophytic microbiota in cereals [10].

The whole grain flour is a rich source of spore-forming bacteria, causing rope-like spoilage of bread. Thus, 45 out of 327 isolated bacterial strains produced amylase and caused rope-like spoilage in the study of Pereira et al. [138]. *B. licheniformis* accounted for 62% of the identified microorganisms, followed by *B. sonorensis* (20%) and *B. cereus* (11%), while *B. pumilus* and *Paenibacillus polymyxa* accounted for only 2% of isolates each.

Fraberger et al. [88] showed inhibition of *B. subtilis*, *B. cereus*, and *B. licheniformis* by most LAB. The antibacterial effect was strain-specific. Different strains of *L. plantarum*, *F. sanfranciscensis*, *Loigolactobacillus coryniformis*, and *L. paracasei* were effective to varying degrees against the studied *Bacillus* species. In other studies, *L. paraplantarum* and *Pediococcus acidilactici* from spontaneous fermentation of einkorn wheat showed high antagonism against *B. subtilis* and *B. cereus* [139], while *W. cibaria* from spontaneous fermentation of chia flour suppressed *B. subtilis* [140]. In turn, Iosca et al. [137] isolated *L. plantarum*, *P. pentosaceus*, and *L. citreum*, which were highly antagonistic against the agents of ropy bread spoilage. The studied LAB had operons for bacteriocins, but phenotypic confirmation of their production was not obtained. All LAB from 12 typical Bulgarian sourdoughs [106] also suppressed bread spoilage by *B. subtilis*. From them, *L. plantarum* 08B217, two strains of *L. brevis* 07B198, and *E. durans* 09B374 showed the highest antagonism with growth inhibition zones of 65–74 mm. However, neutralization of CFS reduced antagonism by 33–52%. At the same time, denaturation of protein compounds by boiling and treatment with trypsin reduced antagonism in almost all LAB strains to an even greater extent. The results indicate that protein antimicrobial compounds make the greatest contribution to the antibacterial activity of LAB, followed by organic acids. Li et al. [141] showed that the use of sourdough inhibits the germination of Bacillus spores in bread, an effect enhanced by reutericyclin produced by *L. reuteri*.

## 4. Contribution of Other Microorganisms to Protecting Bread from Spoilage

### 4.1. Yeast

The yeasts used in sourdough are also capable of inhibiting the growth of mold fungi. The contribution of *Wickerhamomyces anomalus* yeast is most often noted. Thus, in the studies of Pahlavani et al. [142], *W. anomalus* inhibited the growth of *A. flavus* in challenge dough, especially on wheat bread with the addition of spontaneously fermented barley (Type IV sourdough). In the studies of Coda et al. [124], *W. anomalus* yeast also contributed to the antifungal activity of sourdough with LAB by producing ethanol and ethyl acetate. Germination of *P. roqueforti* conidia was not observed at ethanol and ethyl acetate concentrations of 1.69 (36.6 mM) and 6.81 (77.0 mM) mg/mL, respectively. Syrokou et al. [72] also selected 12 out of 195 yeast strains from spontaneously fermented Greek wheat sourdoughs that inhibited the growth of *P. chrysogenum* by producing ethyl acetate. These yeasts were assigned to *S. cerevisiae*, *W. anomalus*, and *Pichia fermentans*. *W. anomalus* was later shown to produce non-protein anti-mold metabolites, including ethanol, ethyl acetate, isoamyl alcohol, isoamyl acetate, benzaldehyde, and 2,4-di-tert-butylphenol [143]. The latter two compounds had the lowest MICs. In turn, the yeast *Pichia kudriavzevii*, which inhibited the growth of *A. niger* and *A. flavus*, was isolated from buckwheat sourdough by Shahryari et al. [144]. There is also evidence of a reduction in mycotoxin levels when using yeast. Thus, compressed baker’s yeast showed better results in reducing ochratoxin levels in bread compared to other forms [145].

### 4.2. Acetic Acid Bacteria

Since AAB are often found in the spontaneous microbiota of sourdoughs, the possibility of using them as part of sourdough microorganisms was investigated. Thus, *Kozakia baliensis* and *Neoasaia chiangmaiensis* were shown to produce polysaccharides in dough from wheat, whole grain, spelt, and rye flours [146]. The polysaccharide content in the fermented dough exceeded 30 g/kg, which will allow the use of one-tenth to one-third of the sourdough in the dough. At the same time, the acetic acid yield in the dough with *K. baliensis* was 2–3 times higher (up to 100 mM/kg) than that of lactobacilli. The obtained data may indicate the prospects of using AAB in sourdoughs to improve the shelf life of bread. This assumption is supported by positive results in preventing mold formation and extending the shelf life of bread when using kombucha as a starter [147,148]. *A. pasteurianus* and *Gluconobacter oxydans* isolated from fermented cocoa pulp-bean mass also revealed promise as sourdough starter cultures [48]. Increased dough acidification when adding AAB to the starter [41] and an increase in acetic acid production [149] may contribute to improved shelf life of bread.

## 5. Synergistic Role of Different Microbial Groups in Sourdough

Natural substrates contain many different microorganisms with their metabolic characteristics. At the same time, microorganisms cohabiting a particular substrate constantly interact with each other directly or remotely. Therefore, the general indicators of a fermented product are not a simple sum of the properties and metabolites of individual community representatives. Cross-feeding, nutritional competition, and quorum sensing determine the dynamics of microorganism interactions in complex substrates [150].

LAB and sourdough yeasts partially compete for nitrogen sources. However, there is ample evidence of mutualistic relationships between yeast and LAB in natural fermentations of various substrates [150,151,152,153]. It was shown that co-cultivation of *P. pentosaceus* OP91 with yeast in rye sourdough increased the total LAB abundance, total titratable acidity, alcohol, and exopolysaccharide contents and significantly reduced pH as compared to separate cultures [154]. Moreover, yeast *P. kudriavzevii* TM26 was more effective than *S. cerevisiae* TM15 and *K. marxianus* TM39. In another study, during solid-state fermentation of wheat gluten, the quantities of yeasts *Saccharomyces boulardii* and *Pichia kluyveri* were increased when co-inoculated with *Latilactobacillus sakei* [155]. In Arena et al. [156] research, the ability of molds to grow on bread surfaces was significantly reduced in dual cultures of different LAB isolates and yeasts, while LAB biodiversity was increased.

LAB are highly auxotrophic and require several amino acids, vitamins, and other nutrients for their growth, which is associated with a protein-rich natural habitat [157]. Accordingly, these microorganisms have a developed system of transporters and enzymes aimed at peptide catabolism. LAB grow poorly on media without available amino acids, peptides, and vitamins (especially group B) [158]. Yeast, in turn, is a good source of B vitamins [159]. It has also been shown that, in a nitrogen-rich environment, *S. cerevisiae* yeast secretes a pool of amino acids consumed by LAB [160]. The peculiarities of yeast metabolism regulation cause excessive secretion of amino acids, creating a stable niche for the growth of LAB.

Gabrielli et al. [161], using 13C-labeling of peptides and extracellular medium, showed the preferential utilization of galactose from LAB by yeast and confirmed the previously reported [160] reverse flow of amino acids from yeast to LAB. Furthermore, this study revealed a more complex cross-exchange of metabolites between LAB and yeast. LAB shared galactose, pyruvic acid, lactic acid, aspartic acid, glutamic acid, and glycine with yeast, while yeast shared mainly amino acids [161]. Moreover, studies by Konstantinidis et al. [162] showed that the co-evolution of L. plantarum and a riboflavin auxotrophic S. cerevisiae strain resulted in LAB acquiring the ability to overproduce riboflavin to support yeast growth and improve the utilization of amino acids secreted by yeast.

The main carbon sources in sourdoughs are maltose, glucose, and maltodextrins. Both yeast and LAB in sourdoughs produce amylolytic enzymes to break down the substrate. The interactions between yeast and LAB regarding the use of the carbon source are poorly understood. However, there is evidence that yeast can supply LAB with glucose and fructose in addition to amino acids [163]. The availability of a nitrogen source for LAB in a flour medium depends on the action of cereal proteases that are active under acidic conditions, since LAB do not have proteinase in the cell wall. Therefore, acidification of the medium is an important factor in continuing growth and obtaining the necessary peptides that can be assimilated by cell wall peptidases. Obtaining amino acids from yeast significantly accelerates LAB growth in the sourdough.

During flour fermentation, ample bound phenolic compounds of cereals are released. Of which the main one (90%) is ferulic acid, and the rest include caffeic, dihydrobenzoic, and sinapic acids [163]. Therefore, the production of derivatives of ferulic and caffeic acids is characteristic of sourdough LAB (Table 1). Thus, the production of antifungal and antibacterial compounds in the sourdough is the result of synergism of both the main microbiota and the substrate itself.

There is evidence that co-cultivation of yeast and LAB in various substrates promotes an increase in antioxidant activity and antimicrobial properties [154]. The studied yeasts showed various types of antioxidant activity involving both the cell wall and intracellular metabolites, and suggesting the presence of several mechanisms: the ability to donate electrons and hydrogen atoms due to non-enzymatic antioxidants such as polyphenols, glutathione or carotenoids; absorption of hydroxyl radicals due to the presence of intracellular antioxidants (glutathione and phenolic compounds); enzymatic neutralization of superoxide anions (superoxide dismutase). Several studies have noted the synergism of antioxidant activity and antimicrobial action of various compounds and components [164,165,166,167]. Also, the total phenolic content positively correlated with the antibacterial activity of essential oils [168]. Antioxidants, for example, polyphenols, can damage target cells indirectly or directly [169,170]. The co-cultivation of LAB and yeast in Lim et al. studies [154] increased the total phenol content and enhanced antioxidant properties of rye sourdough. During storage of rye sourdough inoculated with *B. cereus* for five days, the pathogen titer was reduced by individual cultures of *P. pentosaceus* and the studied yeast by one to three orders of magnitude. The joint culture of *P. pentosaceus* OP91 and *S. cerevisiae* TM15 reduced the pathogen titer by five orders of magnitude [154]. In turn, co-fermentation of sourdough with the bacteriocin producer *L. brevis* LAS129 and yeast *Pichia membranifaciens* YS05 or *Pichia anomala* YS26 increased the amount of LAB while decreasing the CFU of the bread spoilage agent *B. subtilis* ATCC 35421 [171].

Co-cultures of *L. sakei* and *S. boulardii* or *P. kluyveri* produced higher isoamyl acetate levels during solid-state fermentation of wheat gluten [155]. The combination of *L. sakei* and *P. kluyveri* produced significantly higher amounts of 3-methylbutanal, 2-methylbutanal, 3-methylbutanol, 2-methylbutanol, and isoamyl acetate than when inoculated individually. Solid-state fermentation ensured spatial fixation of microorganisms and the absence of nutrient competition. The synergistic metabolism of LAB and yeast in some amino acid transamination promoted the formation of more aldehydes and their subsequent reduction to alcohols and esters. However, a decrease in yeast numbers and isoamyl acetate levels was observed upon switching to liquid-state fermentation, probably due to the inhibition of their growth by accumulated undissociated acetic acid.

In a system consisting of several physiologically and taxonomically distinct groups of microorganisms, their spatial separation by substrate matrix is of great importance, which allows maintaining a high species and metabolic diversity. In a system consisting of LAB, yeast, and AAB, amino acids and vitamins secreted by yeast into the medium promote the growth of LAB and AAB, while carbon dioxide, ethanol, and esters produced by yeast are metabolized by AAB into acetic acid [172]. It is also known that AAB can oxidize various carbohydrates, alcohols, and related compounds with the release of aldehydes, ketones, and organic acids [173,174]. This pathway of incomplete oxidation is called AAB oxidative fermentation. The metabolites released into the environment can suppress other microorganisms and can also be further used by AAB for complete oxidation when carbon sources in the environment are depleted. In addition to acetic acid, the final products of oxidation of the corresponding alcohols by AAB can also be propionic, butyric, isobutyric, pentanoic, hexanoic, glyceric, phenylacetic, 2-chloropropionic, (S)-2-phenyl-1-propionic, 2-methylbutanoic, (R)-2-hydroxybutyric, 3-hydroxypropionic, and other acids [174], which can contribute to the antifungal activity of sourdough during co-fermentation with yeast. At the same time, another characteristic metabolic pathway of AAB is the so-called acetate overoxidation of organic acids. For example, *A. pasteurianus* is able to oxidize lactic acid to acetoin [173]. The conversion of lactic acid to pyruvic acid has also been shown [174]. Simultaneously, it was found that dough obtained using yeast and AAB-based sourdoughs, as well as yeast, LAB, and AAB-started sourdoughs, were characterized by a higher content of essential amino acids [55]. Studies of sourdough microbial communities that also include AAB are rare, although it has been shown that the introduction of AAB into the community of LAB *L. brevis* and yeast *S. cerevisiae* contributes to greater acidification of the sourdough [41]. In the same study, in some cases, yeast disappeared from the community, which may be a consequence of increased acetic acid production. Changes in the profile of volatile compounds in sourdoughs with the addition of AAB [41] may also affect the shelf life of bread, since a significant part of the antifungal compounds are volatile.

Another group of microorganisms sometimes studied for bread protection, together with LAB, are propionic acid bacteria, which convert lactic acid to propionic acid [87]. Thus, in the studies of Ran et al. [175], a mixed culture of *Propionibacterium freudenreichii* D6 and *L. plantarum* L9 produced propionic, lactic, and acetic acids. However, in this study, acetic acid, rather than propionic acid, was predominantly responsible for the antifungal activity against *A. niger*, *P. crustosum*, and *A. flavus*.

The interactions of LAB with yeasts and propionibacteria have been largely characterized. Research on the microbial interactions of AAB in sourdoughs is just beginning. The positive interactions between different genera and species of LAB are also poorly understood. A significant proportion of LAB produce bacteriocins, which are of primary importance in competition with related microorganisms for nutrients. The key to creating artificial LAB co-cultures is metabolic nutrient dependencies that exclude competition and ensure successful interactions [158]. During sourdough maturation, the rate of maltose utilization and the acid tolerance of individual species and strains play an important role in LAB interactions [176]. A study of three proteolytic and three non-proteolytic LAB strains belonging to *Enterococcus faecalis*, *Lactococcus lactis*, and *L. plantarum* in a dairy medium showed that not every proteolytic LAB stimulated non-proteolytic LAB equally [177]. Strong interactions were associated with higher concentrations of tryptophan, valine, phenylalanine, leucine, isoleucine, and peptides. Their use led to greater acidification of the medium and production of volatile compounds. The authors showed that the stronger the interactions between LAB, the more pronounced the functional results can be obtained. Since, as shown in the previous sections, various compounds produced and released by LAB in the sourdough matrix can exert a synergistic effect together, the successful combination of different LAB species and strains is key to exhibiting an antifungal effect.

Delavenne et al. [178] revealed that quorum sensing plays a role in the antifungal activity of LAB. Low molecular peptides can induce certain defense responses in the LAB cell, particularly the production of bacteriocins [179]. In turn, Ström et al. [94] suggested that the antifungal activity of LAB cyclic dipeptides is a secondary effect, and the main reason lies in quorum sensing or other unknown mechanisms. This research group also showed that cyclic dipeptides were synthesized in cells de novo, since the synthetic growth medium did not contain proteins or peptides. The quorum-sensing system of LAB is not yet fully understood. Recent studies have shown that the production of phenyllactic acid, the main antifungal acid of LAB, is mediated by quorum sensing [180].

Another discovery showed that acetate (acetic acid) triggers plantaricin production, acting as an alternative bacteriocin autoinducer in the stationary growth phase of *L. plantarum* [181]. This finding may be useful in more fully revealing the interactions of LAB and AAB in sourdoughs and the possible synergistic antifungal action. At the same time, *S. cerevisiae* reduced the activity of autoinducer-2 of *Enterococcus faecium* in their co-culture [182]. Such intercellular interactions can support co-cultures of LAB and yeast and ensure their mutual existence and significant metabolite production.

On the other hand, the quorum sensing system of *L. paraplantarum* increased plantaricin production in co-culture with *W. anomalus* during food competition [183]. Simultaneously, the yeast released some essential amino acids for the LAB growth. The researchers emphasized the complex and multidirectional nature of synergistic cross-feeding in LAB-yeast co-cultures. In turn, Ameur [184] assumed that the subdominant players in microbial communities cooperate synergistically with dominant species. Thus, the production of compounds with antagonistic activity in co-cultures lies somewhere in the balance between food competition and cross-feeding. The task for further research is identifying the exact mechanisms of such interactions.

## 6. New Strategies for Using Bread Sourdoughs

In recent years, additives of various components that improve the quality and shelf life of sourdough bread have been actively studied. The potential of legumes, pseudo-legume pseudo-cereals, and other additives in the development of new bakery products is noted [21,185]. This area is particularly relevant in connection with the need to ensure a balanced diet and transition to a diet with a higher content of plant proteins to reduce greenhouse gas emissions [185,186,187].

Most often, additives are pre-fermented and used as sourdough in bread making. Sourdoughs based on non-standard flours have not yet been studied much due to their wide variety. In the study of such starters and additives, special attention is paid to increasing the nutritional value and digestibility of the final products, as well as the content of biologically active components in them [188,189,190]. For example, it was shown that pea sourdough increased the digestibility of protein in traditional durum wheat focaccia, reducing starch digestibility [191]. In turn, fermented mixtures of wheat and legume flours (green lentils, fava beans, chickpeas) were characterized by a significant increase in antioxidant activity [192]. The best effect was shown by sprouted lentils fermented with *L. casei* and *Kluyveromyces marxianus*. Increases in antioxidant activity and total phenolic content have been observed with replacement of up to 15% (*w*/*w*) of wheat flour with flour derived from legume seeds (grass pea, yellow lupine, and narrow-leaf lupine) [193], with the addition of 25% (*w*/*w*) yellow pea flour to gluten-free wholemeal rice flour [194], and in various mixtures of wheat flour and legumes [195].

There is evidence of antimicrobial activity in microorganisms isolated from spontaneously fermented substrates. Thus, *W. cibaria* was isolated from spontaneously fermented chia sourdough, inhibiting the growth of *B. subtilis*, *P. roquefortii*, and *A. niger*. *Enterococcus casseliflavus*, *E. faecium*, and *L. lactis* CH179 inhibited the growth of both fungi, and *L. rhamnosus* and other strains of *L. lactis* suppressed the growth of only one mold [141].

A large body of data has revealed the effect of non-standard starters and additives on the preservation of bread. Sourdough based on einkorn, oat, hull-less barley, wheat germ, fermented sprouted mung bean and lentil, soya flour and rice bran, chickpea flour, faba bean flour, quinoa and red lentil, amaranth, pitaya fruit, cornelian cherry, chokeberry and pomegranate juices were used to improve the quality, nutritional value and preservation of bread (Table 2). Among the mechanisms of action studied, organic acids, including phenolic, and antifungal peptides were noted. Similar to the origin of the peptides encrypted into flour gliadin found during wheat flour fermentation, Verni’s study showed the origin of antifungal peptides from vicilin and legumin of faba beans [196].

The use of various food wastes has been recently investigated to recycle waste and by-products and mitigate the impact on the environment, as well as for imparting new properties to bread, namely increased nutritional value and shelf life. Thus, the use of barley rootlets (a by-product of the malting and brewing industry) fermented with *W. cibaria*, *L. citreum*, *L. plantarum*, *L. amylovorus*, and *L. reuteri* significantly slowed down the spoilage of bread [217].

Dopazo et al. [218] showed that the use of 5% (*w*/*w*) whey fermented with LAB *L. plantarum* increased the content of lactic and phenyllactic acids and allowed extension shelf life of bread contaminated with *A. flavus* and *Penicillium verrucosum*. The technological properties of the bread were not significantly changed. In the studies of Luz et al. [219], a higher amount of whey from Mozzarella di Bufala Campana was used (replacement of 50% and 100% water). Naturally contaminated bread with 100% water replaced by whey fermented with *L. plantarum* or *L. ghanensis* was characterized by an increase in shelf life by 15 days compared to the control. The fermentation of LAB whey significantly increased the amount of acetic, hexanoic, and octanoic acids. In breads with complete replacement of water with whey, the content of total phenol compounds and antioxidant activity (in DPPH radical-scavenging assay) increased significantly, especially when fermenting whey with *L. plantarum* compared to *L. ghanensis* and unfermented whey. In another study [220], fermented *L. plantarum* whey was used to prepare pita bread. When inoculating the bread surface with *P. expansum* and *Penicillium brevicompactum*, the shelf life of the bread increased to 8 days, and with natural inoculation to 19 days. However, an untrained taste panel detected no differences between the control and whey-produced pita samples.

The use of edible coatings to protect against microbial spoilage in bread baking is a new trend. Coatings include LAB in some carrier or plant materials with antimicrobial and antioxidant properties. Research by Bartkiene et al. [221] showed that the additional use of apple pomace coatings further extended the shelf life of sourdough bread with *Loigolactobacillus coryniformis*, *L. curvatus*, *Lentilactobacillus farraginis*, and *L. mesenteroides*. In another study, cranberry coating of bread with *P. pentosaceus*, *P. acidilactici*, *L. paracasei*, *L. brevis*, *Lactobacillus plantarum*, and *L. mesenteroides* increased antifungal activity against *Aspergillus fischeri*, *Penicillium oxalicum*, *Penicillium funiculosum*, *Fusarium poae*, *Alternaria alternate*, and *F. graminearum* [222].

Also shown to be highly effective in extending the shelf life of pan breads and muffins is the use of chitosan nanoenzymes of LAB *L. plantarum*, *L. helveticus*, and *L. rhamnosus* in the last stage of fermentation and as a coating after baking [223]. *Streptococcus salivarius* subsp. *thermophilus*, *Lactobacillus delbrueckii* subsp. *bulgaricus*, and *L. acidophilus* were used in studies with alginate and whey, which protected bread from contamination by filamentous fungi of the genera *Aspergillus* and *Penicillium* [224]. The addition of LAB to a pectin-based coating to protect bread from *A. flavus*, *A. niger*, and *P. paneum* was also used by Iosca et al. [225]. *L. plantarum*, *F. rossiae*, and *P. pentosaceus* were sprayed onto the coating surface in this study.

Recent trends based on more detailed studies of sourdough microbiomes include the construction of sourdough communities accounting for the metabolic contributions of individual microorganisms. This is a very new approach attempting to identify dominant, subdominant, and satellite species that can jointly provide gene and transcript redundancy in the sourdough [226]. At present, the theoretical foundations for a new generation of sourdoughs, combining multiple microorganisms that are maximally adapted to specific food matrices, are only just being laid.

## 7. Conclusions

Bread protection from spoilage remains an unsolved problem for humanity at this stage. The desire for clean-label products forces researchers to search for and constantly improve safe ways to protect bread. The use of sourdoughs in protecting bread from spoilage is widely studied, but the huge variety of fermentable substrates and local features of the microorganisms inhabiting them, including those causing spoilage, do not allow us to put an end to these studies. The development of modern research methods reveals new features of the interactions of sourdough microorganisms and the mechanisms of their action. To date, the antimicrobial and especially antifungal effects of lactic acid bacteria in starters have been linked to their production of a wide range of organic acids, both aliphatic and those with hydroxyl and phenyl substituents, as well as benzoic acids, which often act in synergy and promote the hydrolysis of flour proteins to form antifungal peptides. Moreover, such peptides have been identified both in wheat sourdoughs and in starters based on non-traditional substrates, being sequences embedded in specific proteins of various types of flour. However, data on antifungal peptides of sourdoughs based on various types of flour are still very limited; their study is a promising direction. The mechanisms of action of antifungal compounds in sourdoughs are still poorly understood. The influence of various non-traditional additives and substrates on the antimicrobial activity of sourdoughs is widely studied. There are still many gaps in this area due to the wide variety of substrates and their microbiota. A current trend is the use of food waste and by-products for baking. Thus, the use of whey in sourdough fermentation has shown encouraging results. There is also a clear trend to expand the list of used starter microorganisms using non-traditional genera of LAB and yeast, such as *Weissella* and *Wickerhamomyces*, and AAB, which are constant and almost unstudied companions of LAB and yeast in the fermentations they carry out.

The practical use of microbial combinations of different species and genera, obtained from mature spontaneous sourdoughs that have already demonstrated antagonistic activity against bread spoilage agents, can accelerate the selection of active strains and identify their compatibility. A significant increase in the shelf life of bread can also be achieved by adding various additives that increase the nutritional value of bakery products and simultaneously promote the antagonistic activity of sourdough microorganisms.

The selection of a variety of microorganisms that best perform significant functions in dough matrices from different types of flour, taking into account the metabolic contributions of dominant, subdominant, and satellite species, is a completely new and promising direction in the research and development of sourdoughs. Applying such synthetic microbial communities will ensure stability and more fully reveal the potential of sourdoughs to improve not only the nutritional, textural, and sensory qualities of bread, but also its preservation and safety.

## Figures and Tables

**Table 1:** Antifungal organic acids produced by lactic acid bacteria for bread preservation.

Name	Systematic Name	Producing LAB Species	Affected Molds	References
Saturated aliphatic fatty acids				
Formic *	formic	*F. sanfranciscensis*; *L. plantarum* and *F. rossiae*	*A. niger*, *F. graminearum*, *P. expansum*, and *M. sitophila*; *P. roqueforti*	[96,101]
Acetic *	acetic	*F. sanfranciscensis*	*A. niger*, *P. paneum*	[25,92,93]
Propionic *	propanoic	*F. sanfranciscensis*; *L. buchneri* and *L. diolivorans*	*A. niger*, *F. graminearum*, *P. expansum*, and *M. sitophila*; *A. clavatus*, *Cladosporium* spp., *Mortierella* spp. and *P. roquefortii*	[49,101]
Butyric	butanoic	*F. sanfranciscensis*	*A. niger*, *F. graminearum*, *P. expansum*, and *M. sitophila*	[101]
n-Valeric	pentanoic	*F. sanfranciscensis*	*A. niger*, *F. graminearum*, *P. expansum*, and *M. sitophila*	[101]
Caproic *	hexanoic	*F. sanfranciscensis*	*A. niger*, *F. graminearum*, *P. expansum*, and *M. sitophila*	[101]
Capric	decanoic	*L. reuteri*	*A. niger*	[102]
Hydroxy acids and phenyl substituted acids				
Lactic *	2-hydroxypropanoic	*L. acidophilus*, *L. casei*; *W. cibaria*, *L. plantarum subsp. plantarum*, *L. pseudomesenteroides*, *F. sanfranciscensis*, *L. brevis*, and *L. pentosus*	*P. crysogenum*, *P. corylophilum*; *A. flavus*, *A. niger*, *P. expansum*	[73,80]
Hydro-cinnamic	3-phenylpropanoic	*L. amylovorus*	*A. fumigatus*	[103]
Phenyl-lactic *	2-hydroxy-3-phenylpropanoic	*L. plantarum*; *L. bulgaricus*; *L. amylovorus*; *L. reuteri* and, *L. brevis*	*E. repens*, *E. rubrum*, *P. corylophilum*, *P. roqueforti*, *P. expansum*, *E. fibuliger*, *A. niger*, *A. flavus*, *M. sitophila*, and *F. graminearum*; *A. niger* and *P. polonicum*; *F. moniliformis*, *F. graminearum*, *F. verticillioides* and *P. expansum*; environmental molds; *Fusarium culmorum* and environmental molds	[47,76,95,99,104,105]
Phloretic	3-(4-hydroxyphenyl)propanoic	*L. amylovorus*	environmental molds	[47,104]
Hydroxy-phenyl-lactic	2-hydroxy-3-(4-hydroxyphenyl)propanoic	*L. amylovorus*	*A. fumigatus*; environmental molds	[47,103,104]
Hydro-caffeic	3-(3,4-dihydroxyphenyl)propanoic	*L. amylovorus*	*F. culmorum*, environmental molds	[47]
Hydro-ferulic	3-(4-hydroxy-3-methoxyphenyl)propanoic	*L. amylovorus*	*F. culmorum*, environmental molds	[47,104]
2-Hydroxyiso-caproic	2-hydroxy-4-methylpentanoic	*L. amylovorus*, *L. reuteri*, and *L. brevis*	*F. culmorum*, environmental molds	[47]
3-Hydroxy-capric	3-hydroxydecanoic	*L. reuteri*	*A. niger*	[102]
3-Hydroxy-lauric	3-hydroxydodecanoic	*L. reuteri*	*A. niger*	[102]
Unsaturated phenyl-substituted acids				
ρ-Coumaric	(2E)-3-(4-Hydroxyphenyl)prop-2-enoic)	*L. amylovorus*	*A. fumigatus*	[103]
Caffeic	(2E)-3-(3,4-dihydroxyphenyl)prop-2-enoic	*L. plantarum*	*P. expansum*, *P. roqueforti*, *P. camemberti*, *F. moniliformis*, *F. graminearum*, *F. verticillioides*, *A. niger*, and *A. parasiticus*	[99]
Ferulic	(2E)-3-(4-hydroxy-3-methoxyphenyl)prop-2-enoic	*L. brevis*	*F. culmorum*, environmental molds	[47]
2-Methyl-cinnamic	2e-3-2-methylphenylprop-2-enoic acid	*L. amylovorus*	*A. fumigatus*	[103]
Sinapic	3-(4-hydroxy-3,5-dimethoxyphenyl)prop-2-enoic acid	*L. bulgaricus*	*F. moniliformis*, *F. graminearum*, *F. verticillioides*, and *P. expansum*	[99]
Polyphenol compound				
Chlorogenic acid	(1S,3R,4R,5R)-3-{[(2E)-3-(3,4-Dihydroxyphenyl)prop-2-enoyl]oxy}-1,4,5-trihydroxycyclohexane-1-carboxylic acid	*L. plantarum* and *L. bulgaricus*	*P. expansum*, *P. roqueforti*, *P. camemberti*, *F. moniliformis*, *F. graminearum*, *F. verticillioides*, *A. niger*, and *A. parasiticus*	[99]
Dicarboxylic acids				
Azelaic	nonanedioic	*L. amylovorus* and *L. reuteri*	*F. culmorum*, environmental molds	[47]
Uronic acids				
D-Glucuronic acid	(2S,3S,4S,5R,6R)-3,4,5,6-Tetrahydroxyoxane-2-carboxylic acid	*L. amylovorus*	*A. fumigatus*	[103]
Benzoic (aromatic) acids				
Salicylic	2-hydroxybenzoic acid	*L. amylovorus*	*A. fumigatus*	[103]
Gallic	3,4,5-trihydroxybenzoic	*L. plantarum*	*P. expansum*, *P. roqueforti*, *P. camemberti*, *F. moniliformis*, *F. graminearum*, *F. verticillioides*, *A. niger*, and *A. parasiticus*	[99]
Vanillic	4-hydroxy-3-methoxybenzoic	*L. plantarum* and *L. bulgaricus*	*P. expansum*, *P. roqueforti*, *P. camemberti*, *F. moniliformis*, *F. graminearum*, *F. verticillioides*, *A. niger*, and *A. parasiticus*	[99]
Syringic	4-hydroxy-3,5-dimethoxybenzoic	*L. plantarum*	*P. expansum*, *P. roqueforti*, *P. camemberti*, *F. moniliformis*, *F. graminearum*, *F. verticillioides*, *A. niger*, and *A. parasiticus*	[99]

* At least one study has shown the greatest significance of this acid in the manifestation of the antifungal effect among the mixture of acids produced.

**Table 2:** Using sourdoughs with non-standard components.

Component	LAB	Target	Mode of Action	Reference
Einkorn sourdough	*L. paraplantarum* and *P. acidilactici*	*B. subtilis ATCC6633*, *B. cereus ATCC11778*	Not studied	[139]
	*L. crustorum* and *L. brevis*	*Penicillium carneum*, *A. flavus* and *A. niger.*		
Oat-sourdough	*P. pentosaceus*	*A. flavus*	Increased total phenolic content and antioxidant activity	[197]
Hull-less barley sourdough	*P. acidilactici* and *L. plantarum*	*P. carneum*, *A. flavus*, and *A. niger*	Not studied	[198]
Spelt-based sourdough	*W. cibaria* and *P. pentosaceus*	*F. verticillioides*, *A. flavus*	Not studied	[199],
Fermented sprouted mung bean sourdough	*L. brevis*	*A. niger*	Not studied	[200,201]
Fermented sprouted mung bean sourdough	*P. pentosaceus*	*A. niger*	Not studied	[202]
Fermented sprouted lentil with fennel extract	*P. acidilactici*	*A. niger*	Not studied	[203]
20% (*w*/*w*) of rice bran	*L. plantarum*	*Penicillium commune* and *A. flavus*; *aflatoxin*	Lactic and phenyllactic acids	[204]
50% (*w*/*w*) of fermented soya flour and rice bran	*Propionibacterium freudenreichii* and *W. confusa*	*Environmental molds*	Acetic and propionic acids	[205]
Fermented extracts of chickpea, quinoa, and buckwheat flour	*Lactobacillus*spp., *Leuconostoc*spp.	*A. niger*, *A. flavus*, *Penicillium* spp., *Bacillus* spp.	Organic acids	[206]
Fermented chickpea flour	*L. plantarum* and *F. rossiae*	*P. roqueforti*, *P. paneum*, and *P. carneum*	Peptides of 12–20 amino residues	[207]
Fermented faba bean flour (30% *w*/*w*)	*L. brevis*	*P. roqueforti*	Peptides of 11–22 amino acid residues, encrypted into sequences of vicilin and legumin type B; defensin-like protein (8792 Da) and a non-specific lipid-transfer protein (11,588 Da)	[196]
Fermented faba bean flour (15% *w*/*w*)	*L. citreum*	*Potentially antifungal*	Lactic, acetic, 4-hydroxybenzoic, caffeic, coumaric, ferulic, phenyllactic acids	[208]
Fermented quinoa and red lentil supplement	*Enterococcus hirae*	Environmental molds	Not studied	[209]
Fermented amaranth sourdough supplemented with purslane powder	*L. brevis*	*A. niger*	Not studied	[210]
Wheat germ sourdough along with dehydrated spinach puree	*L. lactis*	*A. flavus*	Not studied	[211]
20% (*w*/*w*) of fermented pitaya fruit	*L. plantarum* and *P. pentosaceus*	*A. niger*, *Cladosporium sphaerospermum*, and *P. chrysogenum*	Phenolic acids: gallic, caffeic, protocatechuic; increased antioxidant activity	[212]
Sprouted clover seeds sourdough	*Lacticaseibacillus rhamnosus*	*Aspergillus brasiliensis*	Increased antioxidant activity	[213]
Fermented cornelian cherry supplement	*L. plantarum ATCC 14917*	*Environmental molds*, *Bacillus* spp.	Increased total phenolic content and antioxidant activity; lactic, acetic, formic, n-valeric, and caproic acids	[214]
Fermented chokeberry juice supplement	*L. paracasei*	*Environmental molds*, *Bacillus* spp.	Lactic and acetic acids; increased total phenolic content and antioxidant activity	[215]
Fermented pomegranate juice supplement	*L. plantarum*	*Environmental molds*, *Bacillus* spp.	Increased total phenolic content; lactic acid, acetic acid	[216]

## Data Availability

No new data were created or analyzed in this study. Data sharing is not applicable to this article.
